# Beyond the Norm: Unusual Coexistence of Wilson's Disease and Hypoparathyroidism

**DOI:** 10.7759/cureus.54516

**Published:** 2024-02-20

**Authors:** Rucha Sawant, Pranav Chaudhari, Khadija F Hamdulay, Sunil Kumar, Sourya Acharya

**Affiliations:** 1 Internal Medicine, Jawaharlal Nehru Medical College, Datta Meghe Institute of Higher Education and Research (DMIHER), Wardha, IND; 2 General Medicine, Jawaharlal Nehru Medical College, Datta Meghe Institute of Higher Education and Research (DMIHER), Wardha, IND; 3 Medicine, Jawaharlal Nehru Medical College, Datta Meghe Institute of Higher Education and Research (DMIHER), Wardha, IND

**Keywords:** intact parathyroid hormone, hyperphosphatemia, hypocalcaemia, hypoparathyroidism, wilson’s disease

## Abstract

Wilson's disease (WD) encompasses diverse clinical symptoms involving the liver, nervous system, and kidneys. The fundamental cause of this condition is the build-up of copper in organs, mainly the hepatic and brain parenchyma. Here, we are reporting the hospital presentation of a male patient in his 20s who had been experiencing severe irritability, abdominal pain, distension, and yellowish discoloration of the skin for the previous 75 days. Upon examination of blood pressure, a refractory carpopedal spasm was found in him. In addition to Kayser-Fleischer (KF) rings in his cornea, he exhibited elevated 24-hour urine copper and serum ceruloplasmin (CP). He was diagnosed as a case of WD with a rare association of hypoparathyroidism.

## Introduction

Wilson's disease (WD) is caused by an autosomal recessive mutation in the ATP7B gene located on chromosome 13, which encodes the P-type copper transporter ATPase [1]. The hepatic copper metabolism is disrupted by this mutation, resulting in a reduced excretion of copper by the biliary pathway and reduced ceruloplasmin (CP) synthesis. Consequently, copper deposits in organs such as the hepatic and brain parenchyma. Even though many enzymes depend on copper for proper functioning, an excess can have a destructive effect. The liver is usually the first organ to be involved. As the disease progresses and the brain gets involved, it leads to neuropsychiatric symptoms. Kayser-Fleischer (KF) rings around the cornea are reported in 66% of WD cases [2]. Other uncommon associations are those with renal and cardiovascular systems.

The initial manifestations of hypoparathyroidism are those of hypocalcemia requiring immediate medical intervention. The distinctive features of this uncommon endocrine deficiency condition are low serum magnesium, low serum calcium, elevated serum phosphorus, and abnormally low levels of parathyroid hormone (PTH) in the blood. Out of the many causes of hypoparathyroidism, WD is one of the rarest. Copper deposition in the parathyroid glands reduces the glands' functioning. This leads to patients showing symptoms of hypocalcemia due to hypoparathyroidism. The association of hypoparathyroidism with WD is one of the rarest occurrences reported only a few times in the past [3] and, hence, is a topic for discussion here.

## Case presentation

**Figure 1:**
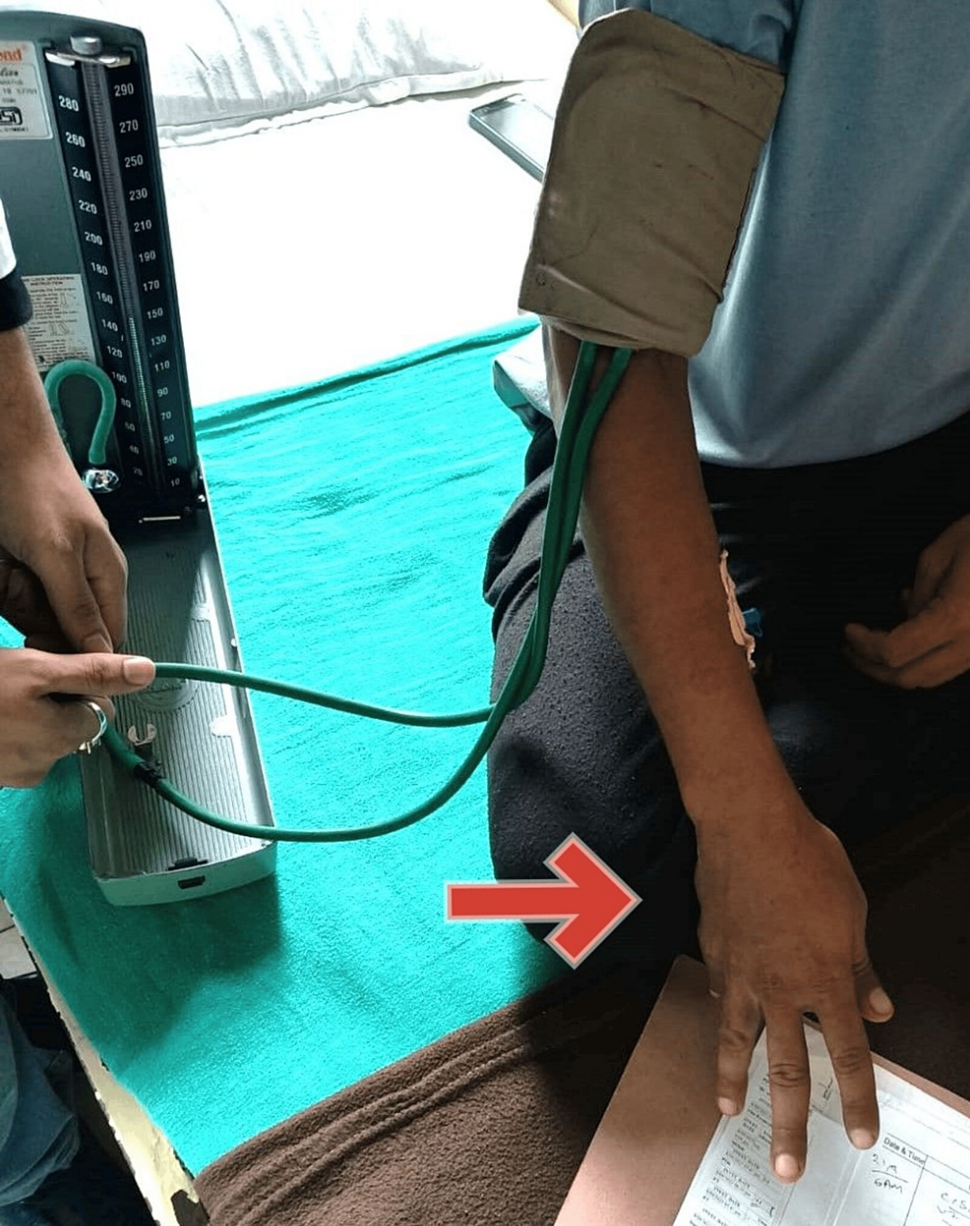
Trousseau's sign

A male in his 20s presented with concerns of frequent irritability, abdominal discomfort, and swelling in the lower limbs over the past two months. These symptoms appeared gradually and had been progressing. Upon further inquiring, he complained of mild abdominal distension and yellowish discoloration of the skin, which had gone unnoticed until the last seven days, when it aggravated. While recording blood pressure, a routine physical examination, the patient's wrist, thumb, and metacarpophalangeal joints went into flexion, and hyperextension of the interphalangeal joints (Figure 1) was observed, suggestive of trousseau's sign, thus warranting further investigation for hypocalcemia.

His laboratory investigations showed reduced serum calcium, magnesium, and intact parathyroid hormone (iPTH) and increased vitamin D and serum phosphate (Table 1).

**Table 1:** Overall laboratory parameters Hb: hemoglobin; MCV: mean corpuscular volume; WBC: white blood cell; INR: international normalized ratio; AST: aspartate aminotransferase; ALT: alanine aminotransferase

Parameters	Patient values on admission	Patient values on discharge	Control values
Hb (g/dL)	8.4	10.2	12-18
MCV	92.3	82.1	81-102
WBC (/mm^3^)	6800	7200	4500-11,000
Total platelet (x10^9^/L)	0.14	0.72	1.4-4.5
INR	1.8	1.21	1.01
Serum ammonia (µmol/L)	66	28	9-30
Urea (mg/dL)	21	20	9-22
Creatinine (mg/dL)	1.0	1.1	0.6-1.25
Sodium (mg/dL)	132	139	137-144
Potassium (mg/dL)	4.3	4.8	3.5-5.2
Magnesium (mg/dL)	1.6	2.1	1.7-2.2
Phosphate (mg/dL)	7.1	5.1	2.5-4.4
Calcium (mg/dL)	6.9	9.3	8.4-10.1
Intact parathyroid hormone (pg/ml)	2.7	5.2	7.5-53.5
Vitamin D (ng/ml)	24.2	34	>30
Total bilirubin (mg/dL)	6	2.9	0.2-1.3
Direct bilirubin (mg/dL)	4	1.8	0-0.3
AST (U/L)	166	88	<50
ALT (U/L)	68	65	17-59
24-hour urinary copper (μg)	88	85	20–50
Serum ceruloplasmin (mg/dL)	11	10	20- 40
Total protein (g/dL)	6.8	6.6	6-8.3
Serum albumin (g/dL)	3.0	3.5	3.4-5.4
Serum ferritin (ng/ml)	17	460	17.5-464
Serum iron (mcg/dL)	38	140	49-181
Direct total iron binding capacity	500	340	261-462
Vitamin B12	15	511	239-913

**Figure 2:**
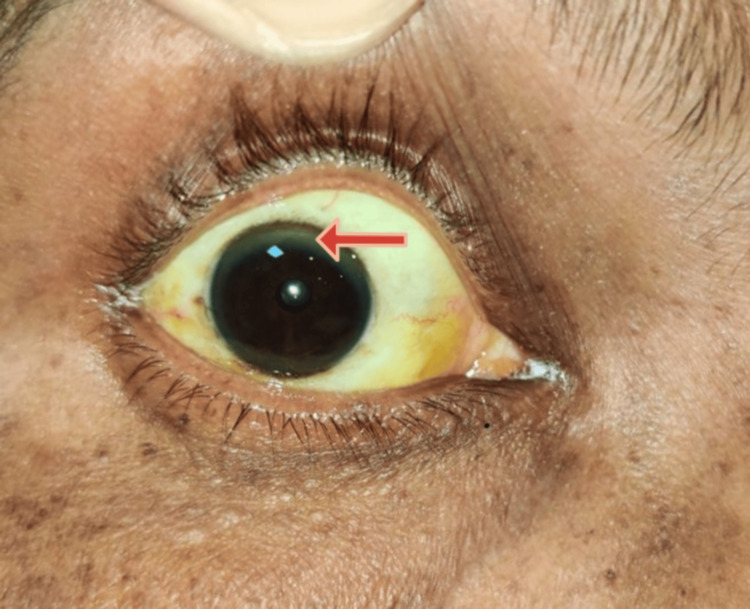
Gross examination showing the KF ring in the right eye KF: Kayser-Fleischer

**Figure 3:**
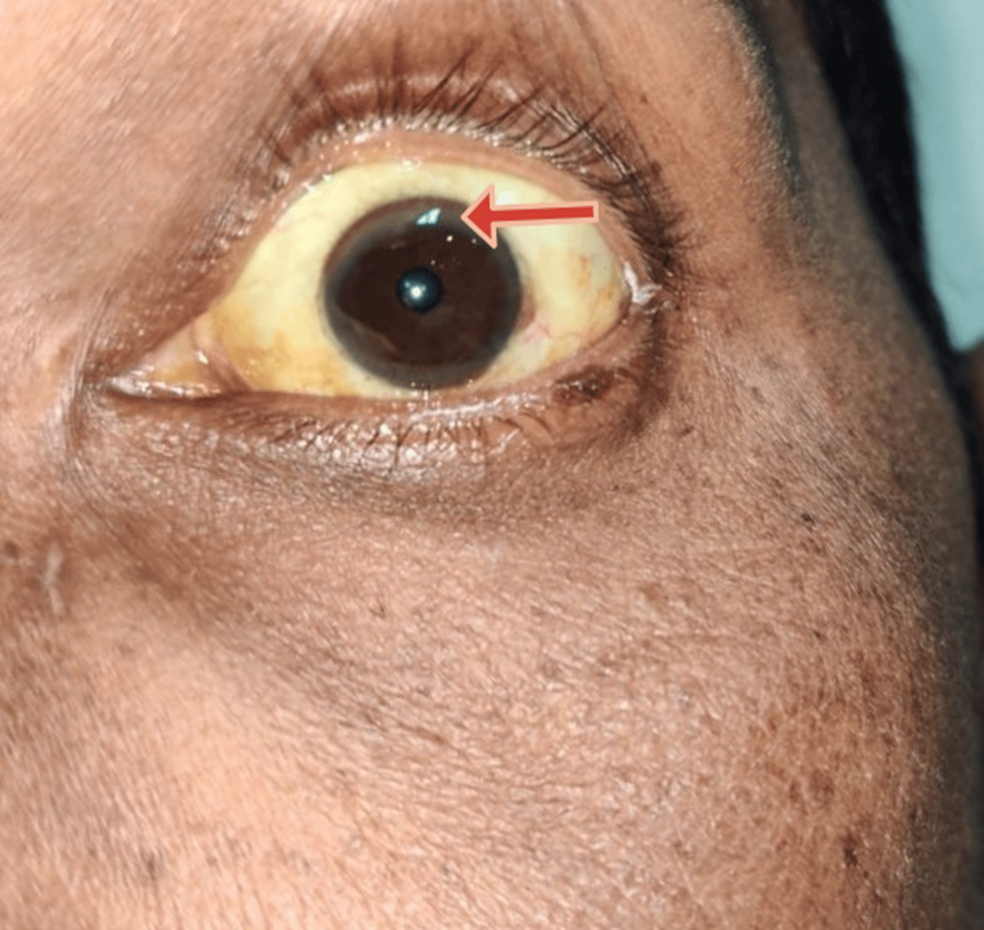
Gross examination showing the KF ring in the left eye KF: Kayser-Fleischer

**Figure 4:**
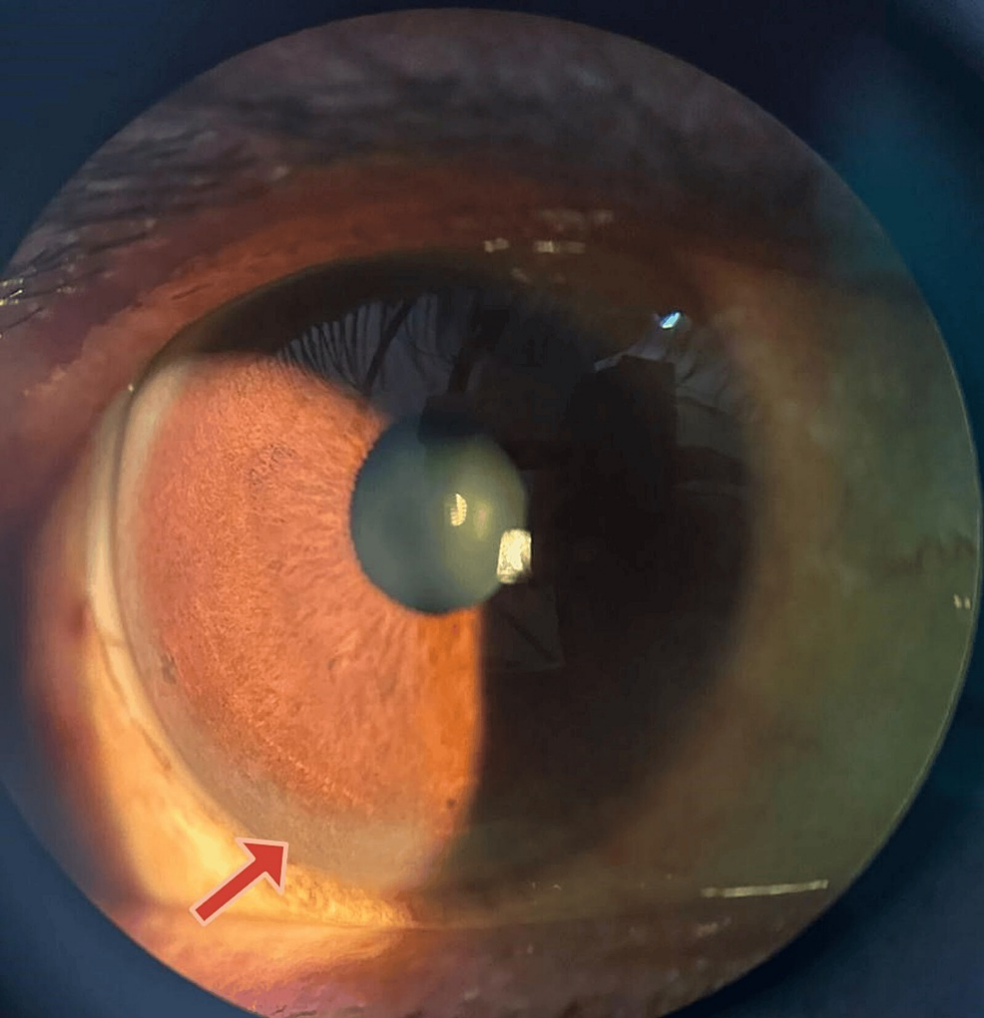
Slit lamp examination showing the KF ring KF: Kayser-Fleischer

99mTc-MIBI dual-phase imaging was performed to rule out parathyroid glands as the offender. As a result of this finding, the diagnosis of hypoparathyroidism was made. Also, the patient's liver function test was deranged, showing increased total bilirubin, direct bilirubin, aspartate aminotransferase (AST), and alanine aminotransferase (ALT). Ultrasonography of the abdomen revealed chronic liver disease, and common etiologies of hepatocellular type of liver injuries, like chronic alcohol intake and viral hepatitis, were ruled out by history taking and a card test, respectively. Autoimmune hepatitis was excluded since antinuclear antibodies and anti-smooth muscle antibodies were found to be negative. Upon further working up the patient, his 24-hour urinary copper was high, and serum CP was low. On gross examination of the eyes (Figure 2, Figure 3), a corneal ring was observed and was confirmed with slit lamp examination to be KF rings on the cornea (Figure 4).

Thus, the patient was diagnosed with WD with hypoparathyroidism. He was supplemented with intravenous calcium gluconate 10 mg 10% wt/vol in 50 ml 0.9% sodium chloride slowly over five minutes and intravenous magnesium chloride 30 mmol/d. His carpal spasms improved, but he had frequent relapses. He was given additional magnesium chloride of 20 mmol/d intravenously, and a repeat calcium gluconate was given at an infusion of 900 mg in 1 L of 0.9% sodium chloride over 24 hours. He was given oral calcitriol 0.5 mcg daily. He was also given D-penicillamine 500 mg/day and zinc for WD and advised to avoid a diet rich in copper, like nuts, mushrooms, and organ meats. Since his iron requirement was 1077 mg, he was also supplemented with parenteral iron sucrose 200 mg daily for five days and intramuscular vitamin B12 1000 µg once daily for seven days. Gradually, his symptoms improved over the next 10 days, and he was discharged.

Following his initial treatment and discharge, the patient returned for a follow-up appointment after 60 days. He reported significant improvement in his symptoms. However, a liver stiffness of 44 kPa was detected on a fibro scan, prompting the recommendation for liver transplantation.

## Discussion

The following case report discusses the diagnosis of a patient with WD, along with the rare association of hypoparathyroidism. Usually, the amount of copper consumed in the diet exceeds the body's daily requirement; hence, most of it undergoes biliary excretion. WD is a hereditary condition that impedes the biliary excretion process, leading to the deposition of copper in organs. The impairment of P-type ATPase, a copper transporter inside hepatocytes, leads to this condition. Copper needs to be transported from proteins that act as chaperones inside cells for both biliary excretion and the production of functional CP [4].

WD has a higher incidence than previously thought; it may be as high as one in every 30,000 [5]. The age of initial occurrence typically falls within the range of 5-35 years old. The male preponderance in India (M:F=2.7:1) is more significantly skewed than the European population (M:F=1.16:1) [6]. KF ring, a classical finding, although not exclusive, is observed in 95% of the cases with neurological symptoms and more than half with liver involvement [5]. KF rings can be visualized using a slit lamp, which reveals a copper-filled corneal Descemet's membrane.

Neurological disease takes as much as 10 years to develop or rarely can be the presenting complaint. The copper deposition in the brain parenchyma happens by multiple mechanisms like oxidative stress, cross-linking of DNA, cell membrane damage, and mitochondrial damage [7]. Neurological involvement can be in the form of posture-dependent tremor, which affects the proximal upper limb, Parkinson-like rigidity and bradykinesia, wing-beating tremors, and dystonia with dysarthria [8]. The most prevalent motor impairments encompass dysarthria, oropharyngeal dystonia, and drooling. Changes in speech and drooling are among the initial neurological symptoms [5]. Sometimes, these patients are misdiagnosed as hepatic encephalopathy. Acute liver failure due to WD occurs mainly in young females (female-to-male ratio of 4:1) [2].

Coombs-negative hemolytic anemia with acute renal failure is an uncommon association with WD. This type of anemia can sometimes be the only initial symptom of WD. Our patient had a negative direct Coombs test. The degree of hemolysis is often consistent with the disease severity. The breakdown of liver cells causes a large amount of stored copper to be released into the circulation, further increasing hemolysis. In a study, 12% of the 220 patients developed hemolysis either as acute episodes or as recurrent ones [2].

Behavioral symptoms are prevalent, with some potentially being the presenting symptoms. In cases of advanced neurological diseases, severe cognitive decline can be seen [9]. Some lesser-known clinical features include chondrocalcinosis, increased urinary calcium and amino acids leading to calcium deposition in the renal parenchyma and tubules, cardiomyopathy, osteoarthritis, and hypoparathyroidism [5].

In most patients, liver function normalizes within two years of treatment, while, on the other hand, neurological symptoms are rarely reversed [5]. According to recent guidelines, all symptomatic patients get treated with chelators (penicillamine or trientine). Other treatments include zinc and vitamin E in neurological patients. At present, a liver transplant is the only rescue therapy for a patient with hepatic involvement in WD. Hepatic or stem cell transplantation is the modality that can become the future modality of treatment that will replace liver transplantation [10].

## Conclusions

This report emphasizes the infrequent association between WD and hypoparathyroidism. While WD has numerous documented clinical correlations, hypoparathyroidism appears rarely within this spectrum. Therefore, any WD patient presenting with severe hypocalcemia warrants thorough evaluation for potential hypoparathyroidism. This might involve various blood tests, imaging studies, and functional assessments to differentiate between intrinsic hypoparathyroidism and one due to WD.
